# Ubiquitin D Correlates with Disease Severity and T Cell Infiltration in Cholestasis: Evidence from Integrated Bioinformatics and Experimental Analyses

**DOI:** 10.7150/ijms.122687

**Published:** 2026-01-01

**Authors:** Huiwen Wang, Jian Zhang, Xun Huang, Shifang Peng

**Affiliations:** 1 Infection Control Center, Xiangya Hospital, Central South University, Changsha, China.; 2 Department of Infectious Diseases, Xiangya Hospital, Central South University, Changsha, China.

**Keywords:** UBD, primary biliary cholangitis, primary sclerosing cholangitis, obstructive cholestasis, integrated bioinformatics, T cell

## Abstract

Cholestasis is a complex pathophysiological syndrome characterized by impaired bile secretion and excretion. Extensive research has revealed that Ubiquitin D (UBD) plays a pivotal role in numerous types of malignancies and benign diseases. However, the underlying involvement of UBD in cholestasis remains unclear. The aim of this study was to analyze the role of UBD in Cholestasis. Transcriptome data from cholestasis patients (GSE61260, GSE159676 and GSE183754) were obtained from the Gene Expression Omnibus (GEO) database. These datasets, along with cholestatic mouse models and clinical specimens, were utilized to evaluate hepatic UBD expression. Subsequently, immunohistochemistry and immunofluorescence staining were applied to validate the expression and localization of hepatic UBD. Moreover, the association of hepatic UBD levels with clinical parameters was evaluated using Pearson's correlation analysis. In addition, Gene set enrichment analysis (GSEA) and xCell were employed to investigate the immune-related signatures and immune cell infiltration link to UBD in cholestasis, with findings further validated through immunofluorescence staining. Finally, the association of the hepatic UBD with T cell-related chemokine and chemokine receptor was explored using Pearson's correlation analysis. The results showed that UBD was significantly and consistently upregulated in the livers across cholestasis patient transcriptomic data, experimental mouse models, and clinical specimens. Hepatic UBD levels positively correlate with the severity of cholestasis and hepatocytes are identified as the primary source of UBD in cholestatic livers. Functional enrichment analysis indicated that immune-related pathway was significantly activated in the cholestatic liver with high expression of UBD group. Moreover, hepatic UBD expression was positively associated with the infiltration of T cells and with the expression of T cell-related chemokines and chemokine receptors in cholestasis. In conclusion, UBD is a key gene associated with disease severity and T cells infiltration in cholestasis. These findings provide new insight into the key biomarker of cholestasis and further highlight that UBD might be a promising novel therapeutic target for patients with cholestasis.

## Introduction

Cholestasis is a complex clinical syndrome characterized by the excessive accumulation of intrahepatic bile acids (BAs), leading to inflammatory response-mediated liver injury that can progress to liver fibrosis, cirrhosis, and even liver failure^1^. Cholestasis results from diverse etiologies and manifests in various clinical settings, including primary biliary cholangitis (PBC), primary sclerosing cholangitis (PSC), viral hepatitis, cholelithiasis, and other liver diseases^2^. In the current clinical practice, only ursodeoxycholic acid (UDCA) and obeticholic acid (OCA) have been authorized by the FDA for the treatment of limited types of cholestasis; however, their therapeutic efficacy and safety are less than satisfactory^3^-^5^. Therefore, it is worth deeply exploring the molecular mechanism underlying the pathological process of cholestasis which may provide therapeutic targets to combat this complex disease.

Ubiquitin D (UBD), a ubiquitin-like protein, directly targets substrates and mediates ubiquitin-independent proteasomal degradation^6^, ^7^. UBD is widely expressed across multiple organs and regulates diverse cellular processes, including signal transduction, cell cycle progression, apoptosis, autophagy, and immune responses^8^, ^9^. Recently, the up-regulation of UBD in numerous types of malignancies^10^-^13^ and other chronic benign disease^14^-^17^ has attracted increasing attention. In the field of liver disease, elevated UBD expression drives tumor cell invasion, metastasis^18^, and chemoresistance in hepatocellular carcinoma (HCC)^19^, ^20^. Additionally, hepatic UBD exacerbates metabolic dysfunction-associated steatotic liver disease (MASLD) by disrupting lipid metabolism. It is reported that the UBD expression is up-regulated by pro-inflammatory stimuli IFN-γ and TNFα^21^, while the induced UBD can further promote inflammation^22^, ^23^. Although inflammation is a well-recognized hallmark of cholestasis and emerging evidence suggests a potential link between UBD and hepatic inflammatory responses^24^, ^25^, the precise role of UBD in cholestasis remains to be elucidated.

Microarray and high-throughput sequencing technologies offer powerful tools for identifying genetic alterations and functional pathways in disease. Here, we reanalyzed transcriptomic data from the GEO database to evaluate UBD expression in cholestatic liver tissues across different etiologies. Subsequently, the hepatic expression of UBD was validated in the cholestasis mouse models and clinical liver samples. Meanwhile, the correlation of hepatic UBD levels with the severity of cholestasis in patients was analyzed. Moreover, Gene Ontology (GO) and Kyoto Encyclopedia of Genes and Genomes (KEGG) analyses were conducted to elucidate the biological differences between UBD-high and UBD-low groups. Finally, the immune profiling and experimental validation with clinical liver tissues were performed to evaluate the associations between UBD expression and immune cell infiltration. This work aimed to reveal the potential role of UBD in the progression of cholestatic liver disease, which may provide novel therapeutic targets for patients with cholestasis.

## Materials and Methods

### Data acquisition and identification of differentially expressed genes

Liver transcriptome datasets (GSE61260^26^, GSE159676^27^, and GSE183754^28^) were obtained from the Gene Expression Omnibus (GEO) database (https://www.ncbi.nlm.nih.gov/geo/). Differentially expressed genes (DEGs) were identified using either the “limma” or DESeq2 package in R, with statistical significance defined as an adjusted p-value < 0.05 and | log2 fold change (FC) | ≥ 1.

### Functional enrichment analysis

Gene Set Enrichment Analysis (GSEA v4.3.3) was performed to evaluate whether predefined gene sets exhibited statistically significant and concordant differences between biological states. Reference gene sets were obtained from the Molecular Signatures Database (MSigDB v7.5.1). Functional enrichment analysis was assessed using the KEGG (C2) and Gene Ontology (GO, C5) subsets, with significance thresholds set at a false discovery rate (FDR) < 25% and a nominal p-value < 0.05.

### Analysis of immune cell infiltration

The xCell algorithm was applied to transcriptomic data to analyze possible associations between the UBD mRNA levels and immune cell infiltration in cholestatic liver tissues^29^.

### Animal experiments

Eight-week-old male C57BL/6 mice were purchased from the Hunan Slake Jingda Laboratory Animals Co., Ltd. All mice were housed under specific pathogen-free (SPF) conditions with an environment of 12-hour light/dark cycle, 50 ± 10% relative humidity and a temperature of 22 ± 2°C. Mice were assigned randomly into three groups as follows: (1) the control group in which mice received only a standard chow diet without intervention; (2) the 0.1% 3,5-diethoxycarbonyl-1,4-dihydrocollidine (DDC) diet group in which mice fed a normal diet supplement with 0.1% DDC; (3) the bile duct ligation (BDL) group in which mice underwent surgical BDL procedure. All mice were euthanized after two weeks of experimentation and a portion of liver tissue was fixed in 10% neutral-buffered formalin for histological assessment while the remaining tissues were snap-frozen in liquid nitrogen and stored at -80°C for subsequent molecular analyses. All animal procedures were approved by the Institutional Animal Care and Use Committee of Xiangya Hospital, Central South University (No.202504063).

### Immunohistochemical and immunofluorescence analysis

The experimental procedures for immunohistochemical staining were performed according to established protocols^5^. Briefly, the paraffin-embedded liver sections were deparaffinized and hydrated. Antigen retrieval was achieved with Tris-EDTA buffer (pH 9.0). Following the block of endogenous peroxidase activity, sections were incubated overnight at 4°C with a primary antibody targeting UBD (1:500 dilution; Proteintech, cat#13003-2-AP, China). Detection was achieved using DAB chromogen (ZSBio, cat#SP-9000, China) with hematoxylin counterstaining. Bright-field images were acquired using a Leica DMIL LED microscope (200× magnification; Leica Microsystems, Germany).

Immunofluorescence staining assays were performed with multiplex Immunofluorescence staining kits (Abiowell, cat#AWI0690, China) according to the manufacturer's instructions. The liver sections were incubated primary antibodies, including anti-UBD antibody (1:100 dilution), anti-CD3 antibody (1:50 dilution; Santa Cruz, cat# sc-20047, USA), anti-ALB antibody (1:100 dilution; Abiowell, cat#AWA11256, China), anti-CK19 antibody (1:100 dilution; Abcam, cat#ab52625, USA), anti-aSMA antibody (1:100 dilution; Abcam, cat# ab124964, USA) at room temperature for 60 min. After washing, sections were probed with HRP-conjugated IgG at RT for 30 min and reacted with fluorophore-conjugated tyramine molecules for 15 min. Nuclear counterstaining was performed using DAPI for 5 minutes. Fluorescence images were captured on a ZEISS LSM880 confocal microscope (200× magnification; Carl Zeiss AG, Oberkochen, Germany). Quantitative analysis was performed on three randomly selected fields per section using Image-Pro Plus 6.0 software (Media Cybernetics, Rockville, USA). The immunoreactive area was calculated as the percentage of positive staining relative to the total tissue area.

### RNA extraction and RT-qPCR analysis

Total RNA was extracted from mouse liver tissues using TRIzol reagent (Invitrogen, USA). Complementary DNA (cDNA) was then synthesized from the extracted RNA using the PrimeScript RT reagent kit (TaKaRa, Japan) according to the manufacturer's protocol. Quantitative PCR (qPCR) amplification was performed using SYBR Green Premix Ex Taq (Takara, Japan) on a Light Cycler 480 system (Roche, Switzerland). Relative gene expression quantification was determined through the comparative Ct (2-ΔΔCt) approach, with GAPDH serving as the reference gene. The primers employed were as follows: GAPDH, forward, aggtcggtgtgaacggatttg; GAPDH, reverse, tgtagaccatgtagttgaggtca; UBD, forward, ccaatggcggttaatgacctt; UBD, reverse, tttcgatggggcttgaggatt.

### Western blot analysis

Hepatic proteins were extracted from mouse liver samples using RIPA buffer supplemented with protease inhibitors (Roche, USA). Protein quantification was performed using a BCA assay. For immunoblotting, 30 μg of protein per sample was separated by 10% SDS-PAGE gels and transferred onto nitrocellulose membranes. The membranes were blocked with 5% non-fat milk in TBST for 30 minutes and then incubated overnight at 4°C with primary antibodies, including anti-UBD (1:1000 dilution) and anti-GAPDH (1:1000 dilution; Abcam, cat# ab181602, USA). Following HRP-conjugated secondary antibody incubation for 1h at room temperature (RT), protein bands were visualized using enhanced chemiluminescence (ECL; Santa Cruz, USA) and quantified using ImageJ software. GAPDH served as the loading control for UBD normalization.

### Patients and tissue samples

Paraffin-embedded liver sections were obtained from Xiangya Hospital (Changsha, Hunan, China) between 2017 and 2025. The cohort included samples from 7 healthy control (HC), 8 patients with PBC, 4 with PSC and 9 with obstructive cholestasis (OC). Diagnoses of PBC, PSC, and OC were established by experienced hepatologists through comprehensive clinical evaluation, including assessment of symptoms, biochemical markers, radiology, and histopathological examination of biopsied liver tissues. The control liver sections were derived from histologically normal liver tissues without pathological alterations. The detailed clinical characteristics of the enrolled patients are listed in Supplementary Table S1. This study was approved by the Ethical Committee and Institutional Review Board of Xiangya Hospital Central South University (No.2025060952). Written informed consent was obtained from all participants.

### Statistical analysis

Quantitative data are presented as mean ± SD. All statistical analyses were performed using GraphPad Prism (version 9.1.1). Bivariate correlations were assessed using Pearson's correlation coefficient. For intergroup comparisons, we employed two-tailed independent Student's t-tests for normally distributed data and Mann-Whitney U tests for non-normally distributed variables. A significance threshold of p<0.05 was considered statistically significant.

## Results

### UBD is an up-regulated DEGs in the livers of cholestasis

**Figure 1:**
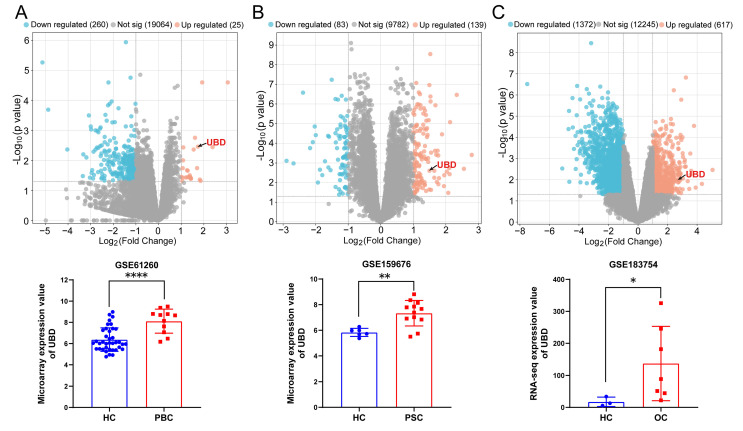
The expression changes of UBD between cholestasis and HC samples based on the GEO dataset. Volcano map of DEGs and the expression value of UBD in cholestasis related GEO dataset including GSE61260 (A), GSE159676 (B) and GSE183754 (C). *p < 0.05, **p < 0.01, ***p < 0.001. UBD, Ubiquitin D; HC, healthy control; PBC, Primary biliary cholangitis; PSC, Primary sclerosing cholangitis, OC; Obstructive cholestasis; DEGs, differentially expressed genes; GEO, Gene Expression Omnibus.

Herein, we assessed the gene expression of UBD in three cholestasis-related datasets from GEO database. The GSE61260 dataset contains 11 liver tissue samples of PBC patients and 38 normal liver tissues of HC. The GSE159676 dataset includes 12 PSC samples and 6 HC samples. The GSE183754 dataset contains 3 OC samples and 7 HC samples. As the volcano map of DEGs and the expression analysis of UBD presented in Fig. 1, UBD is a notably elevated DEG in the liver of cholestasis with different etiology including PBC (Fig. 1A), PSC (Fig. 1B), and OC (Fig. 1C), suggesting its potential role as a common pathogenic factor in cholestasis.

### Validation of UBD in cholestasis mouse models

**Figure 2:**
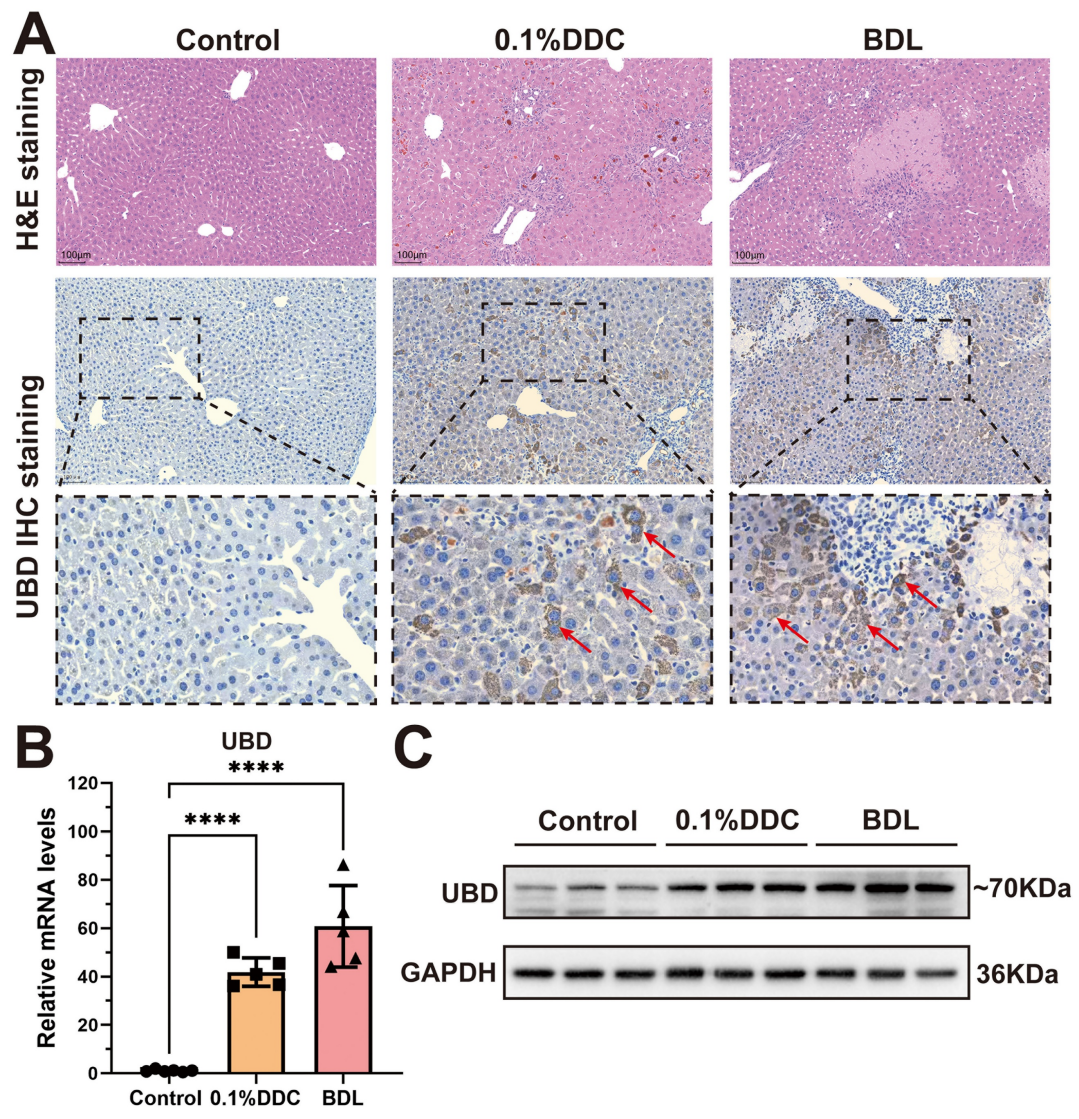
Histological staining and expression analysis of UBD from control and cholestatic mouse livers. (A) Representative H&E staining and UBD IHC staining images of mouse livers from control and cholestasis mice (including 0.1%DDC and BDL). (B) RT-qPCR analyses of UBD mRNA levels in livers of control and cholestasis mice. (C) Western-blot analyses of UBD protein levels in livers of control and cholestasis mice. ****p < 0.0001. UBD, Ubiquitin D; IHC, Immunohistochemistry; H&E, Hematoxylin and eosin; DDC, 3, 5-Diethoxycarbonyl-1,4-dihydroxychollidine. BDL, Bile duct ligation.

To validate UBD expression in cholestatic livers, we constructed two well-established mouse models of cholestasis: the 0.1% DDC diet-induced model which mimics the PSC pathology, and the BDL model that induces OC. H&E staining showed the marked hepatic inflammation in both models, with the distinct porphyrin deposition in the DDC model and characteristic necrotic lesions in the BDL model (Fig. 2A). immunohistochemical staining revealed that UBD protein was rarely expressed in healthy livers but highly elevated in cholestatic livers (Fig. 2A). Consistent with these findings, both qPCR and Western blot demonstrated that the mRNA and protein expression of UBD were significantly increased in the liver of cholestasis mouse model compared to controls (Fig. 2B & C).

### Intrahepatic UBD expression correlated with disease severity of cholestasis patients

**Figure 3:**
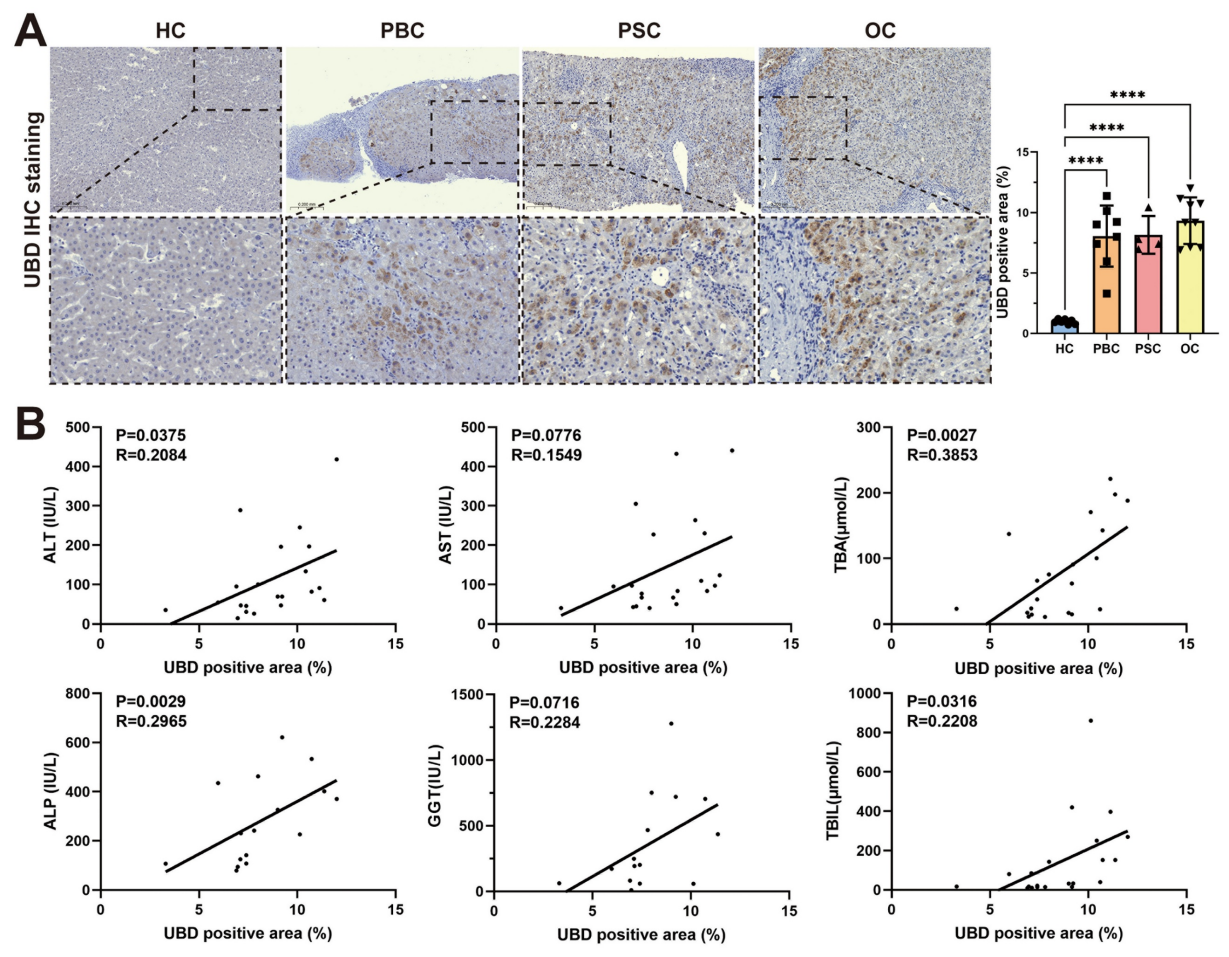
Validation of the intrahepatic expression of UBD and analysis of the correlation of UBD with the severity of cholestasis in patients. (A) Representative UBD IHC staining images of mouse livers from HC and patients with cholestasis including PBC, PSC and OC. (B) The relationship between intrahepatic UBD expression and clinicopathological characteristics in patients with cholestasis. ****p < 0.0001. UBD, Ubiquitin D; IHC, immunohistochemistry; HC, healthy control; PBC, primary biliary cholangitis; PSC, primary sclerosing cholangitis; OC, obstructive cholestasis; ALT, alanine aminotransferase; AST, aspartate aminotransferase; TBA, total bile salts; ALP, alkaline phosphatase; GGT, gamma-glutamyl transferase; TBIL, total bilirubin.

To evaluate the expression of UBD in patients with cholestasis, liver tissue samples of 7 HCs, 8 patients with PBC, 4 patients with PSC and 9 patients with OC were collected. The clinical characteristics of study subjects were summarized in Table 1. Consistent with the findings in mouse models, the IHC staining confirmed significantly elevated UBD protein levels in all three cholestatic conditions (PBC, PSC, and OC) compared to healthy controls (Fig. 3A). Notably, the intrahepatic UBD levels were markedly associated with the clinicopathological features. Regression analysis demonstrated significant positive correlations between UBD expression and serum levels of ALT (R = 0.2084, P = 0.0375), ALP (R = 0.2965, P = 0.0029), TBA (R = 0.3853, P = 0.0027) and TBIL (R = 0.2208, P = 0.0316) (Fig. 3B). These results indicated that UBD is a consistently upregulated protein in human cholestasis and the expression levels of UBD is significantly associated with the severity of cholestasis.

### UBD is specifically expressed in the hepatocytes of cholestatic livers

**Figure 4:**
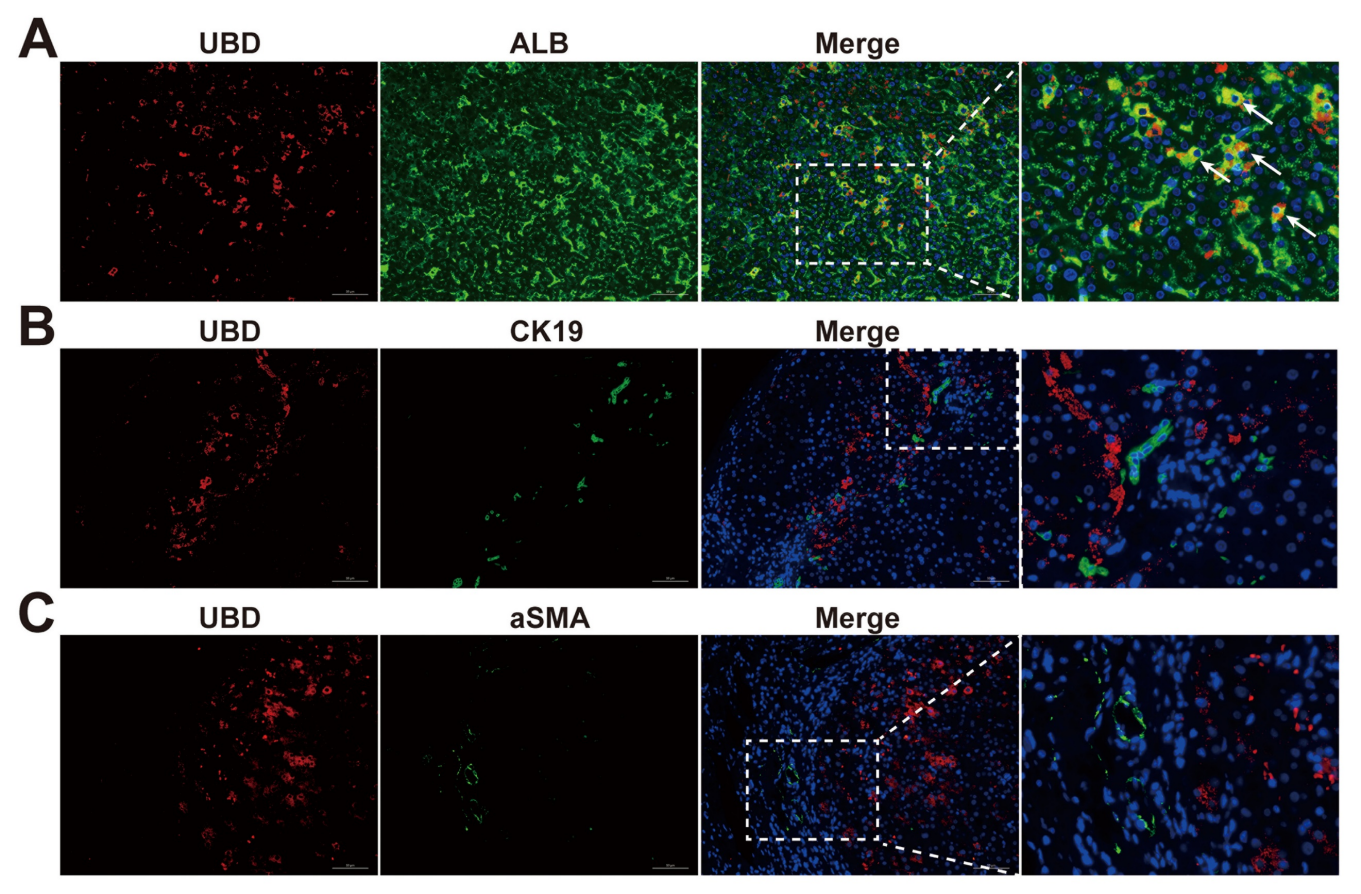
Localization of UBD in the livers of cholestasis patients. (A)Representative images of immunofluorescence staining for UBD (red) and ALB (green) in the liver sections from patients with cholestasis. (B)Representative images of immunofluorescence staining for UBD (red) and CK19 (green) in the liver of cholestasis. (C)Representative images of immunofluorescence staining for UBD (red) and aSMA (green) in the cholestatic liver of patients. Nuclei were labeled with DAPI (blue). UBD, Ubiquitin D; ALB, albumin; CK19, cytokeratin 19; a-SMA, α-smooth muscle actin.

To further explicit the expression pattern of UBD in the different cell types of cholestatic livers, the immunofluorescence staining on liver sections of cholestasis patients was performed. Co-localization studies with albumin (ALB) demonstrated predominant UBD expression in hepatocytes (Fig. 4A). In contrast, UBD was virtually undetectable in non-parenchymal cell populations, including hepatic stellate cells (α-SMA-positive; Fig. 4B) and cholangiocytes (CK19-positive; Fig. 4C). These findings establish hepatocytes as the primary source of UBD in the liver of cholestasis.

### Elevated UBD expression associated with enhanced immune cell activity in cholestasis

**Figure 5:**
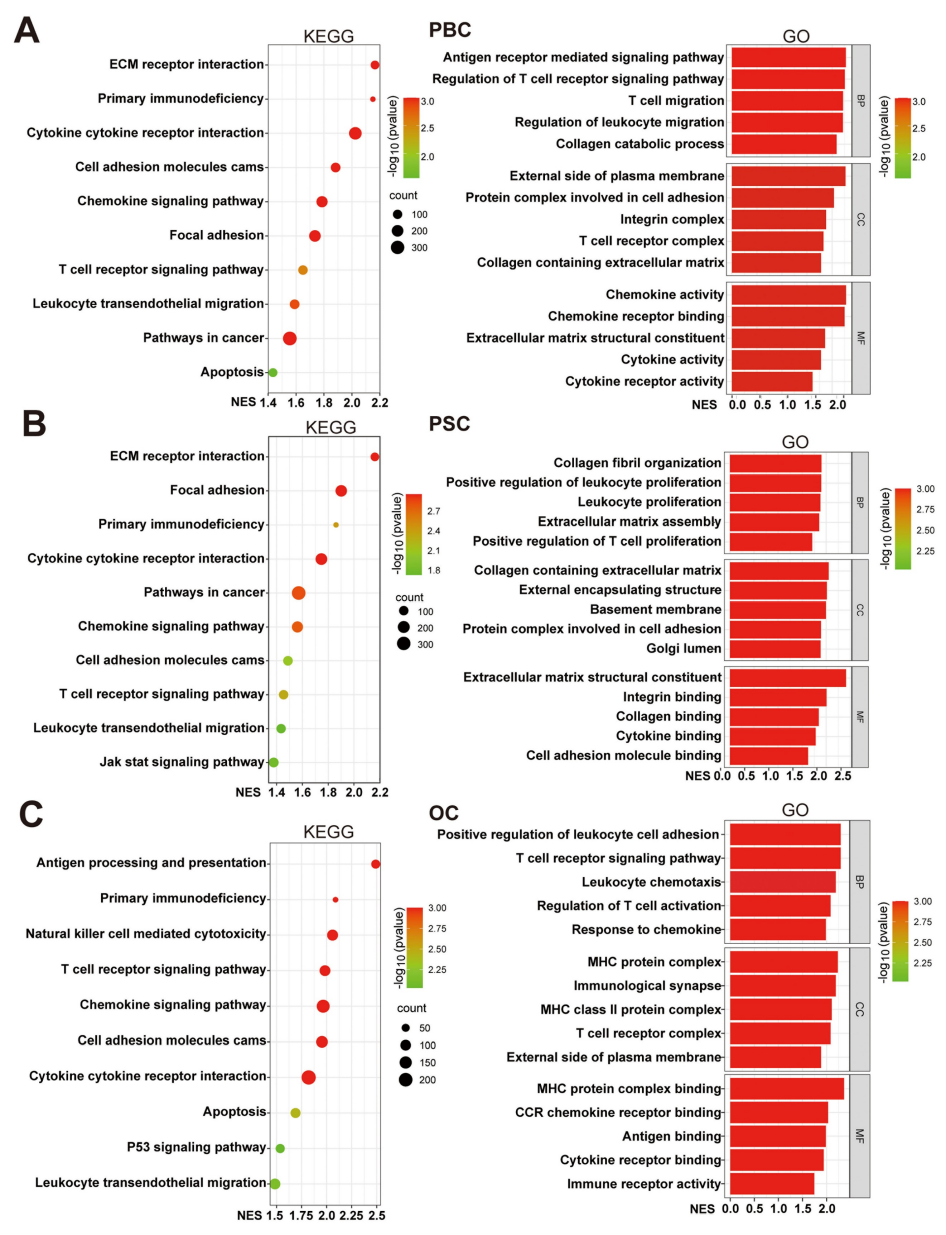
Functional enrichment analysis of cholestasis with high UBD levels. Cholestasis samples in the datasets were divided into UBD low and high-expressed groups based on the median expression of UBD. KEGG and GO pathways analysis of the UBD high-expression group in GSE61260 (A), GSE159676 (B) and GSE183754 datasets (C). UBD, Ubiquitin D; KEGG, kyoto encyclopedia of genes and genomes; GO, gene ontology; NES, normalized enrichment score; BP, biological process; CC, cellular components; MF, molecular function.

To investigate the potential functions of UBD in cholestasis, we stratified cholestasis samples from three datasets into UBD low- and high-expression groups based on median UBD levels. GSEA mediated enrichment analysis was performed to identify the differences of crucial pathways and biological functions between two groups. KEGG pathway analysis demonstrated consistent enrichment of six immune-related pathways in high-UBD groups across all datasets, including the primary immunodeficiency, cytokine-cytokine receptor interaction, cell adhesion molecules cams, chemokine signaling pathway, T cell receptor signaling pathway and leukocyte transendothelial migration (Fig. 5A&B). GO analysis further showed that nearly all significantly enriched biological processes in high-UBD groups were related to immune cell activity, including migration, proliferation, chemotaxis, activation, and adhesion (Fig. 5A&B). These findings indicated that hepatocyte-derived UBD in cholestasis is significantly associated with the enhanced biological activity of immune cells.

### Hepatic UBD expression associated with T cell infiltration in cholestatic livers

**Figure 6:**
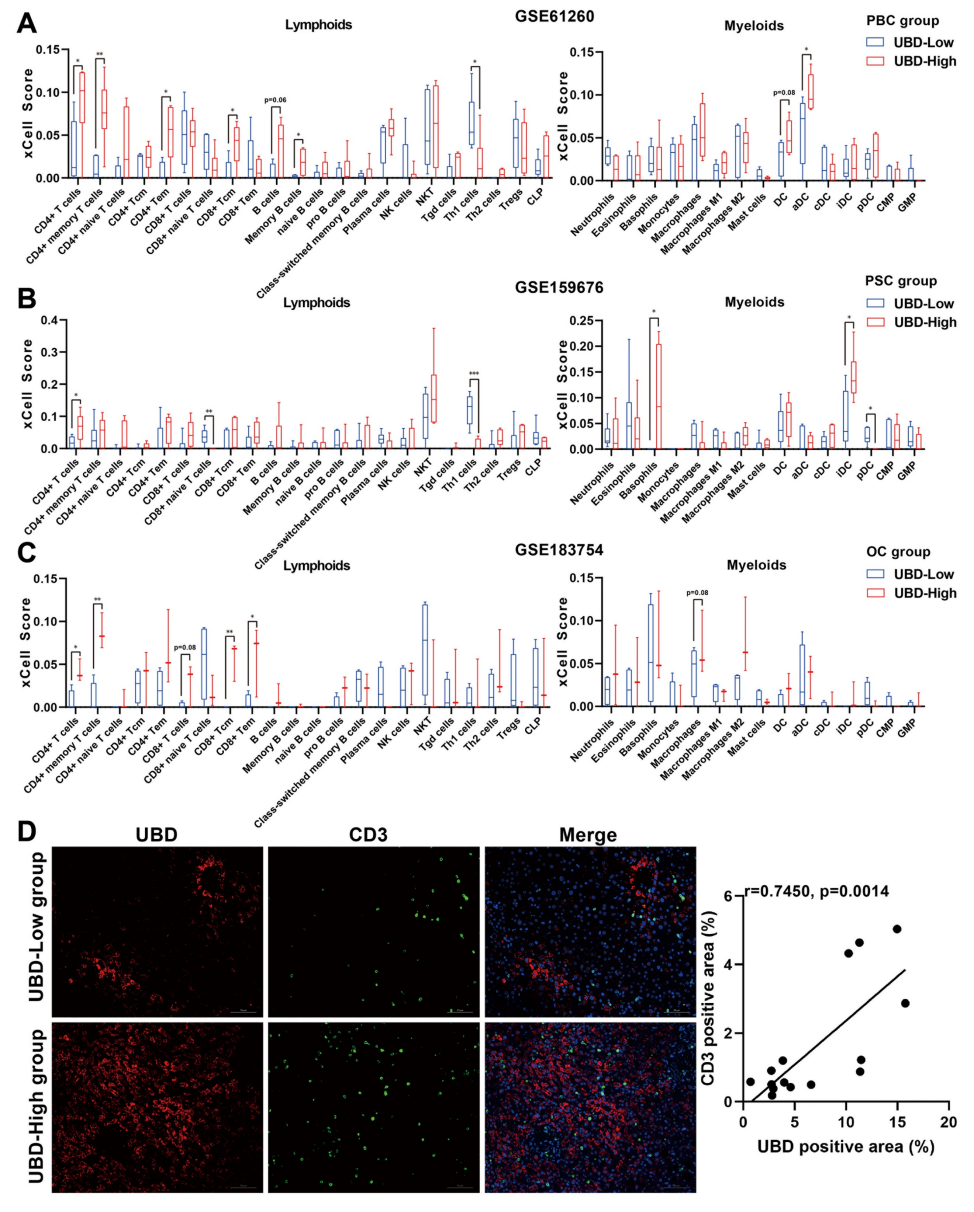
Relationship between hepatic UBD expression and immune cell infiltration in cholestatic livers. Infiltration of lymphoid cells and myeloid cells into cholestatic livers of the low- and high UBD groups according to xCell analysis GSE61260 (A), GSE159676 (B) and GSE183754 datasets (C). (D) Representative immunofluorescence staining images and correlation analysis of UBD and CD3-positive area in livers of cholestasis patients. *p < 0.05, **p < 0.01, ***p < 0.001. UBD, Ubiquitin D.

To investigate the potential impact of hepatic UBD expression on immune cell infiltration during cholestasis, the xCell analysis was performed to compare immune cell densities between UBD low- and high-expression groups across three datasets. In the PBC cohort, the high-UBD group showed significantly increased infiltration of CD4+ T cells, CD4+ memory T cells, CD4+ effector memory T (Tem) cells, and activated dendritic cells (Fig. 6A). The PSC cohort demonstrated elevated levels of CD4+ T cells, basophils, and immature dendritic cells in high-UBD samples (Fig. 6B). Similarly, the OC cohort exhibited increased infiltration of CD4+ T cells, CD4+ memory T cells, CD8+ central memory T (Tcm) cells, and CD8+ Tem cells in the high-UBD group (Fig. 6C). Take above together, the T cells including CD4+ and/or CD8+ T cells were the major intersecting immune cell types that highly infiltrated in the UBD high-expressed group of three datasets.

To further verify the association between hepatic UBD expression and T cell infiltration, the immunofluorescence staining for the liver samples of cholestasis patients was performed. Consistent with our bioinformatic findings, the liver tissues with higher UBD expression were accompanied by more infiltration of T cells (Fig. 6D). Collectively, these results demonstrate that increased hepatic UBD expression is associated with enhanced T cell infiltration in the livers of cholestasis.

### Hepatic UBD levels correlated with T cell-related chemokine and chemokine receptor expression in cholestasis

**Figure 7:**
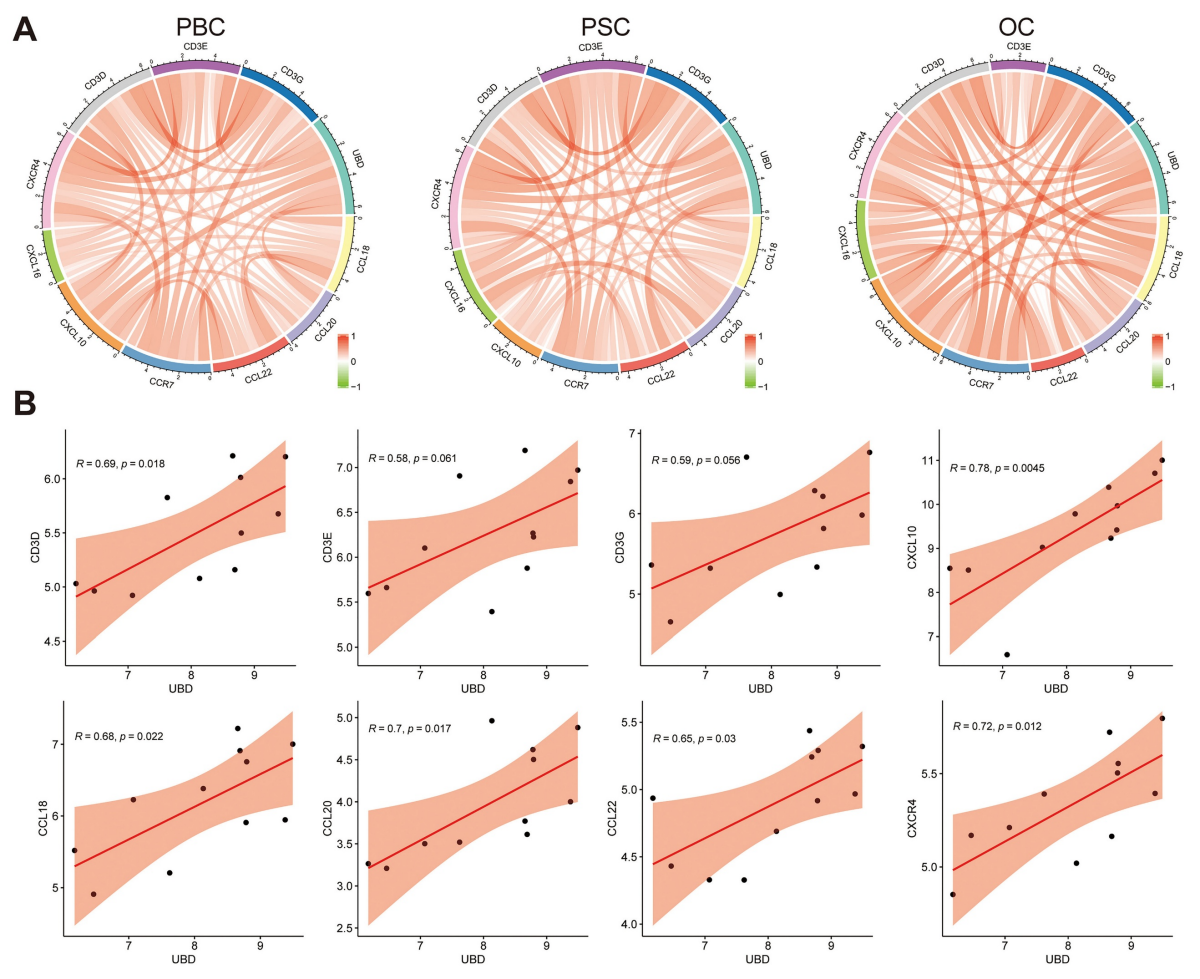
Relationship of UBD expression with the levels of chemokines and chemokine receptors in cholestatic livers of the datasets. (A) The circus diagram depicts Pearson correlations between UBD levels and the expression of chemokines and chemokine receptors in GSE61260, GSE159676 and GSE183754 datasets. (B) Positive correlation of UBD levels with the expression of chemokines and chemokine receptors, including CD3D, CD3E, CD3G, CXCL10, CCL18, CCL20, CCL22 and CXCR4 in GSE61260. UBD, Ubiquitin D.

Given that T cell infiltration in cholestatic liver is mediated by chemotactic signals, we investigated potential associations between UBD expression and T cell-related chemokines/chemokine receptors. As shown in Fig. 7A, correlation analyses across PBC, PSC, and OC datasets revealed significant positive relationships between hepatic UBD expression and multiple T cell-associated chemokines (CCL18, CCL20, CCL22, CXCL10, CXCL16) as well as chemokine receptors (CCR7, CXCR4), in addition to T cell receptor subunits (CD3D, CD3E, CD3G). Specifically, in the PBC cohort, UBD levels showed statistically significant correlations with CD3D, CD3E, CD3G, CXCL10, CCL18, CCL20, CCL22, and CXCR4 expression (Fig. 7B). The PSC cohort demonstrated significant correlations between UBD and CD3D, CD3E, CD3G, CXCL16, CCL20, CCL22, CCR7, and CXCR4 (Supplementary Fig. 1A). Similarly, the OC dataset revealed significant UBD correlations with CXCL10, CXCL16, CCL22, and CXCR4 (Supplementary Fig. 1B). These findings collectively demonstrate that hepatic UBD expression positively correlates with key T cell-recruiting chemokines and their receptors, suggesting a potential mechanism for UBD-mediated T cell migration during cholestasis.

## Discussion

In the present study, through an integrated approach combining bioinformatic analysis and experimental validation, we have identified hepatic UBD as a novel key gene associated with the severity and T cell infiltration of cholestasis across multiple etiologies. The novel findings are summarized as follows: (1) Hepatic UBD expression was markedly up-regulated in both patients (PBC, PSC, and OC) and experimental mouse models (BDL and DDC-induced cholestasis); (2) Hepatic UBD expression was positively correlated with the severity of cholestasis; (3) Hepatic UBD were positively associated with the inflammation activation, especially T-cell infiltration in cholestasis. These results established UBD as a functionally significant mediator in cholestasis and highlighted its potential as a promising therapeutic target for cholestasis intervention.

As a member of the ubiquitin-like modifier (ULM) family, UBD classically functions to target proteins for degradation by the 26S proteasome, with UBD being degraded along with its substrates. Elevated UBD expression has been implicated in promoting mitotic non-disjunction and chromosomal instability, suggesting a potential role in tumorigenesis. Current studies have revealed significant UBD upregulation in various cancers and non-cancer diseases^17^, ^30^-^32^. In liver disorders, the increased hepatic UBD expression shows positive correlations with lipid accumulation in MASLD^33^, enhanced invasiveness and immunosuppression in HCC^18^, ^19^, stellate cell activation during liver fibrosis^34^, and Mallory-Denk body formation in alcoholic steatohepatitis (ASH)^35^. Notably, our study provides the first evidence linking elevated hepatic UBD expression with both the severity of cholestasis and T-cell infiltration in cholestatic liver. Nevertheless, the precise function and underlying mechanisms of the upregulated UBD in the cholestasis warrant further investigation through comprehensive *in vitro* and *in vivo* studies.

Although the etiology of cholestasis varies considerably, the accumulation of bile acids in the liver represents a common pathological feature and serves as the first strike of the cholestatic liver^36^. Excessive toxic bile acids trigger hepatocytes apoptosis and inflammatory factor secretion, promote cholangiocytes proliferation, and stimulate hepatic stellate cells activation - all of which collectively drive liver injury and fibrosis progression in cholestasis^37^. While the immune mechanisms underlying cholestasis have gradually been revealed and highlighted, they remain incompletely understood. Emerging evidence suggests that activation of resident and infiltrating immune cells, along with amplified immune responses, acts as the second hit that exacerbates cholestatic liver injury^38^. Neutrophils infiltrate during the acute phase of cholestasis^36^, while other innate immune cells including macrophages, natural killer cells, and dendritic cells also participate in the cholestatic liver injury^38^, ^39^. Notably, adaptive immune cells - especially activated T cells - accumulate abundantly in cholestatic liver^40^ and promote disease progression through cytokine release and inflammatory signaling cascade activation^38^, ^41^. CD8+ cytotoxic T lymphocytes (CTLs) are widely regarded as the primary effector cells responsible for direct bile duct epithelial cell (BEC) injury, especially in PBC. These autoreactive CD8+ T cells recognize specific autoantigens on BECs and induce apoptosis through mechanisms like perforin/granzyme pathways^42^-^44^. Concurrently, CD4+ T helper (Th) cells orchestrate the inflammatory microenvironment^45^, ^46^. The Th17 subset, in particular, has been identified as a critical pathogenic driver, especially in PSC^47^. Th17 cells, often activated by microbial-derived products from the gut, secrete pro-inflammatory cytokines such as IL-17A, which not only promote neutrophilic inflammation but also contribute directly to fibrogenesis by activating hepatic stellate cells^48^-^50^. Conversely, the persistence of this immunopathology is often linked to a failure in regulatory mechanisms. Regulatory T cells (Tregs), which are essential for maintaining self-tolerance, are frequently found to be numerically deficient or functionally impaired in the cholestatic liver microenvironment^51^, ^52^. Evidence suggests that a high bile acid milieu may destabilize the Treg phenotype, potentially skewing them towards a pathogenic Th17 profile, thus exacerbating the Th17/Treg imbalance^53^. This sustained, multifaceted T cell activation—spanning direct cytotoxicity, pro-inflammatory cytokine secretion, and pro-fibrotic signaling—is critically dependent on the chemokine gradients that recruit these subsets to the portal tracts.

It is reported that UBD induction during the maturation of the dendritic cells enhances the capacity of antigen presentation and T cell stimulation, suggesting its importance in regulating T cell activity^54^. Furthermore, elevated UBD expression correlates with increased immune cell infiltration (including T cells) in skin cutaneous melanoma^55^. In our study, we observed significant UBD induction during cholestasis (Fig. 1-3), with positive correlations to T cell infiltration (Fig. 6), as well as the expression of T cell-related chemokines and their corresponding receptors (Fig. 7). The above findings uncovered the previously unrecognized role of UBD in cholestasis. Besides, our immunofluorescence staining revealed that UBD was specifically expressed by hepatocytes rather than other nonparenchymal cells (Fig. 4). As the disorder of bile acid homeostasis initiates cholestatic liver injury, whether accumulated bile acid triggers hepatic UBD expression remains to be determined.

Several limitations should be acknowledged in this study. Firstly, further efforts are warranted for deeper mechanistic investigations of UBD in the development and progression of cholestasis. Secondly, the potential utility of UBD as a circulating biomarker for cholestasis remains to be validated through systematic analysis of serum UBD levels in patient cohorts. Nonetheless, the findings of our study were likely reliable, as they were based on multiple datasets from patients with cholestasis, as well as experimental verification in clinical liver samples and mouse models of cholestasis with different etiologies.

## Conclusion

In summary, our study identified UBD as a key gene that correlated with both cholestasis severity and hepatic T cell infiltration. These findings reveal previously unrecognized immunomodulatory functions of UBD in cholestatic liver injury, positioning it as a promising novel therapeutic target for cholestasis management.

## Supplementary Material

Supplementary figure and table.

## Figures and Tables

**Table 1:** Clinical Features of Patients

Clinical Features	HC	PBC	PSC	OC
Total samples (Male/Female)	7(2/5)	8(1/7)	4(2/2)	9(4/5)
Age (years)	53.0 ± 2.0	54.0 ± 4.8	52.0 ± 10.8	58.0 ± 4.8 *
ALT (*IU/L*)	11.7 ± 3.1	83.1 ± 68.9 *	54.9 ± 46.9	162.4 ± 125.8 *
AST (*IU/L*)	17.4 ± 2.3	120.9 ± 81.2 *	67.4 ± 28.2 *	197.9 ± 160.1 *
ALP (*IU/L*)	79.9 ± 9.7	340.2 ± 174.6 *	147.8 ± 66.4	267.6 ± 185.8 *
GGT (*IU/L*)	14.1 ±13.2	442.1 ± 444.7	226.0 ± 187.2	306.9 ± 273.6 *
TBA (*μmol/L*)	5.1 ± 4.1	97.4 ± 65.9 *	40.0 ± 36.4 *	78.6 ± 82.8 *
TBIL (*μmol/L*)	9.1 ± 2.3	167.3 ± 285.0	74.1 ± 101.8	155.4 ± 166.4 *
DBIL (*μmol/L*)	3.2 ± 1.5	89.3 ± 130.4	45.6 ± 69.1	92.0 ± 99.8 *

Values are means ± SD. *P < 0.05. Comparisons between groups were made using the two-tailed Student's t-test. Abbreviations: HC, healthy controls; PBC, primary biliary cholangitis; PSC, primary sclerosing cholangitis; OC, obstructive cholestasis; ALT, alanine aminotransferase; AST, aspartate aminotransferase; ALP, alkaline phosphatase; GGT, gamma-glutamyl transferase; TBA, total bile salts; TBIL, total bilirubin; DBIL, direct bilirubin.

## Abbreviations

UBD: Ubiquitin
GEO: Gene Expression Omnibus
GSEA: Gene set enrichment analysis
BAs: Bile acids
HC: healthy control
PBC: Primary biliary cholangitis
PSC: Primary sclerosing cholangitis
OC: Obstructive cholestasis
UDCA: Ursodeoxycholic acid
OCA: Obeticholic acid
HCC: Hepatocellular carcinoma
MASLD: Metabolic dysfunction-associated steatotic liver disease
KEGG: Encyclopedia of Genes and Genomes
DEGs: Differentially expressed genes
GO: Gene Ontology
NES: normalized enrichment score
BP: biological process
CC: cellular components
MF: molecular function
FDR: False discovery rate
SPF: Specific pathogen-free
DDC: 0.1% 3,5-diethoxycarbonyl-1,4-dihydrocollidine
BDL: Bile duct ligation
ULM: ubiquitin-like modifier
ASH: Alcoholic steatohepatitis
IHC: Immunohistochemistry
H&E: Hematoxylin and eosin
ALT: Alanine aminotransferase
AST: Aspartate aminotransferase
TBA: Total bile salts
ALP: Alkaline phosphatase
GGT: Gamma-glutamyl transferase
TBIL: Total bilirubin
ALB: Albumin
CK19: Cytokeratin 19
a-SMA: α-smooth muscle actin
