# Supplementary material for: Ubiquitin D Correlates with Disease Severity and T Cell Infiltration in Cholestasis: Evidence from Integrated Bioinformatics and Experimental Analyses
Source: Int J Med Sci. 2026 Jan 1;23(1):350–62. doi: 10.7150/ijms.122687 (PMC12702096; doi:10.7150/ijms.122687)
Supplement: Supplementary file 1 — Supplementary figure and table. [file ijmsv23p0350s1.pdf]

Supplementary Material

Ubiquitin D Correlates with Disease Severity and T Cell Infiltration in Cholestasis: Evidence from Integrated Bioinformatics and Experimental Analyses

Huiwen Wang[1], Jian Zhang[2], Xun Huang[1]*, Shifang Peng[2]*

1 Infection Control Center, Xiangya Hospital, Central South University, Changsha, China

2 Department of Infectious Diseases, Xiangya Hospital, Central South University, Changsha, China

*** Correspondence:**

Shifang Peng

sfp1988@csu.edu.cn

Xun Huang

huangxun@csu.edu.cn

Supplementary Table 1. The detailed clinical characteristics of patients for Immunohistochemistry analyses

ID	Diagnosis	Gender, Age	ALT (IU/L)	AST (IU/L)	ALP (IU/L)	GGT (IU/L)	TBIL (µmol/L)	DBIL (µmol/L)	TBA (µmol/L)	UBD IHC Score
HC (1)	LM	F, 50y	9.6	15.9	68.9	13.5	10	2.8	1.2	0.93
HC (2)	LM	M, 48y	10	16.9	N.D.	N.D.	10.9	6.4	7.4	0.73
HC (3)	LM	M, 49y	10.1	19	N.D.	N.D.	9.5	2.1	7.4	0.96
HC (4)	IPL	F, 56y	12.6	14.6	83	46.2	3.9	1.5	0.9	0.77
HC (5)	LM	F, 50y	9.6	15.9	68.9	13.5	10	2.8	1.2	1.17
HC (6)	HH	F, 55y	11	17.6	93.8	27.1	8.3	4.2	4.9	1.2
HC (7)	LM	F, 41y	18.8	22	84.8	15.1	11.1	2.9	12.7	1.07
PBC (1)	PBC	F, 54y	245	263.7	226.4	57.8	860	396.7	170.7	10.13
PBC (2)	PBC	F, 55y	35.4	40.4	107	62.2	17	7.9	23.3	3.30
PBC (3)	PBC	F, 55y	54.3	95.2	435.1	171.6	80.8	59.7	137.4	5.97
PBC (4)	PBC	F, 63y	69.4	83.7	621.2	720.7	32.6	19.2	90.8	9.23
PBC (5)	PBC	M, 50y	60.6	123.5	401.6	435.2	152	117	197.7	11.37
PBC (6)	PBC	F, 57y	30.6	66.8	141.7	59.3	21	8.6	66.2	7.4

PBC (7)	PBC	F, 48y	69.4	66.8	326.6	1278.1	32	19.5	17.2	9.00
PBC (8)	PBC	F, 50y	100	227.3	462	751.8	142.9	86	75.7	8.00
PSC (1)	PSC	F, 55y	26	40.3	241.4	466.4	14.3	5.3	10.9	7.80
PSC (2)	PSC	F, 53y	45.7	77	107.5	201.8	14.7	7.8	37.5	7.40
PSC (3)	PSC	M, 35y	133.7	109.3	N.D.	N.D.	250.4	165.3	100.3	10.43
PSC (4)	PSC	M, 65y	14.2	42.9	94.5	9.7	16.9	4.1	11.4	6.97
OC (1)	OC	M, 55y	90.9	97.4	N.D.	N.D.	397.1	247.4	221.4	11.13
OC (2)	OC	F, 51y	47	44.7	231.3	193	10.7	3.4	14.3	7.13
OC (3)	OC	F, 64y	95.2	97.3	78.9	81.8	9.6	4	17.3	6.90
OC (4)	OC	F, 54y	196.8	230.1	N.D.	N.D.	39.7	24.2	22.7	10.60
OC (5)	OC	M, 58y	196.2	432.3	N.D.	N.D.	419.7	227.2	61.9	9.17
OC (6)	OC	M, 62y	81.7	83.6	532.8	703.9	151.9	89.7	142.8	10.73
OC (7)	OC	M, 65y	289	305.1	125.1	248.7	84.8	41.8	23.9	7.10
OC (8)	OC	M, 55y	46.7	50.2	N.D.	N.D.	15.1	9.9	14.9	9.17
OC (9)	OC	F, 58y	418.3	440.8	370	N.D.	269.8	180.1	188.2	12.00

HC, healthy control; LM, liver metastases; IPL, inflammatory pseudotumor of the liver; HH, hepatic hemangioma; PBC, primary biliary cholangitis; PSC, primary sclerosing cholangitis; OC, obstructive cholestasis; ALT, alanine aminotransferase; AST, aspartate aminotransferase; ALP, alkaline phosphatase; GGT, gamma-glutamyl transferase; TBA, total bile salts; TBIL, total bilirubin; DBIL, direct bilirubin; UBD, Ubiquitin D; IHC, immunohistochemistry; M, male; F, female. N.D., not detected.

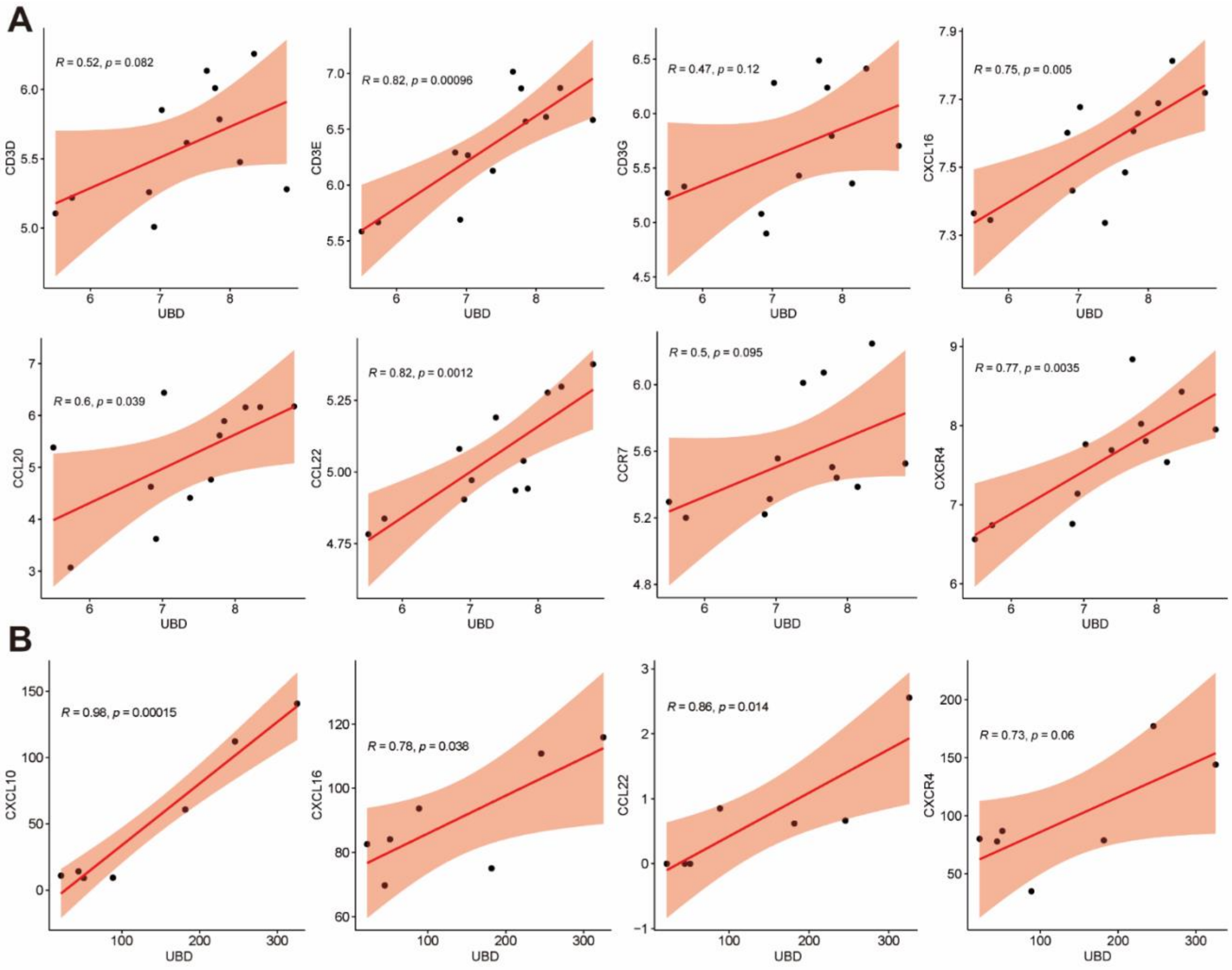

Supplementary Fig. 1. Relationship of UBD expression with the levels of chemokines and chemokine receptors in cholestatic livers of the datasets. (A) Positive correlation of UBD levels with the expression of CD3D, CD3E, CD3G, CXCL16, CCL18, CCL20, CCL22, CCR7 and

CXCR4 in GSE159676. (B) Positive correlation of UBD levels with the expression of CXCL10, CXCL16, CCL22, and CXCR4 in GSE183754. UBD, Ubiquitin D.
